# The proposed systemic thermogenic metabolites succinate and 12,13-diHOME are inversely associated with adiposity and related metabolic traits: evidence from a large human cross-sectional study

**DOI:** 10.1007/s00125-019-4947-5

**Published:** 2019-07-15

**Authors:** Senthil K. Vasan, Raymond Noordam, Mahasampath S. Gowri, Matthew J. Neville, Fredrik Karpe, Constantinos Christodoulides

**Affiliations:** 1 Oxford Centre for Diabetes, Endocrinology and Metabolism, Radcliffe Department of Medicine, University of Oxford, Churchill Hospital, Oxford, OX3 7LE UK; 2 MRC Lifecourse Epidemiology Unit, University of Southampton, Southampton General Hospital, Hampshire, UK; 3 Department of Internal Medicine, Section of Gerontology and Geriatrics, Leiden University Medical Center, Leiden, the Netherlands; 4 Department of Biostatistics, Christian Medical College, Vellore, Tamil Nadu 632001 India; 5 NIHR Oxford Biomedical Research Centre, OUH Foundation Trust, Oxford, UK

**Keywords:** Brown adipose tissue, 12,13-DiHOME, Human, Insulin resistance, Obesity, Succinate

## Abstract

**Aims/hypothesis:**

Circulating succinate and 12,13-dihydroxy-9Z-octadecenoic acid (12,13-diHOME) were recently shown to promote brown adipocyte thermogenesis and protect against metabolic disorders in rodents. This study aimed to evaluate the associations between plasma levels of these metabolites and adiposity and metabolic profile in humans.

**Methods:**

Fasting plasma succinate and 12,13-diHOME levels were quantified using ultra HPLC-tandem MS in 2248 individuals (50% female, mean age 41.3 ± 5.9 years, mean BMI 26.1 ± 4.6 kg/m^2^) in addition to fasting plasma biochemistry. Total and regional adiposity were assessed with dual-energy x-ray absorptiometry. An age- and sex-adjusted linear regression model was used to determine the associations between succinate and 12,13-diHOME levels and body composition and metabolic profile. Two-sample Mendelian randomisation was used to assess the associations between genetically determined BMI and metabolic traits with circulating plasma succinate and 12,13-diHOME.

**Results:**

A one-SD higher plasma succinate and 12,13-diHOME concentration was associated with a 0.15 SD (95% CI 0.28, 0.03) and 0.08 SD (0.15, 0.01) lower total fat mass respectively. Additionally, a one-SD higher plasma 12,13-diHOME level was associated with a 0.09 SD (0.16, 0.02) lower fasting plasma insulin and 0.10 SD (0.17, 0.04) lower plasma triacylglycerol. In Mendelian randomisation analyses, genetically determined higher BMI, fasting hyperinsulinaemia and elevated lipid levels were not associated with changes in either plasma succinate or plasma 12,13-diHOME concentrations. No indications of bias due to directional pleiotropy were detected in the Mendelian randomisation analyses.

**Conclusions/interpretation:**

Our findings tentatively suggest that plasma succinate and 12,13-diHOME may play a role in the regulation of energy metabolism and brown adipose tissue activation in humans. Further studies encompassing direct assessment of brown adipose tissue activity and dietary supplementation are necessary to investigate the potential beneficial effects of these metabolites on systemic metabolism.

**Electronic supplementary material:**

The online version of this article (10.1007/s00125-019-4947-5) contains peer-reveiwed but unedited supplementary material, which is available to authorised users.

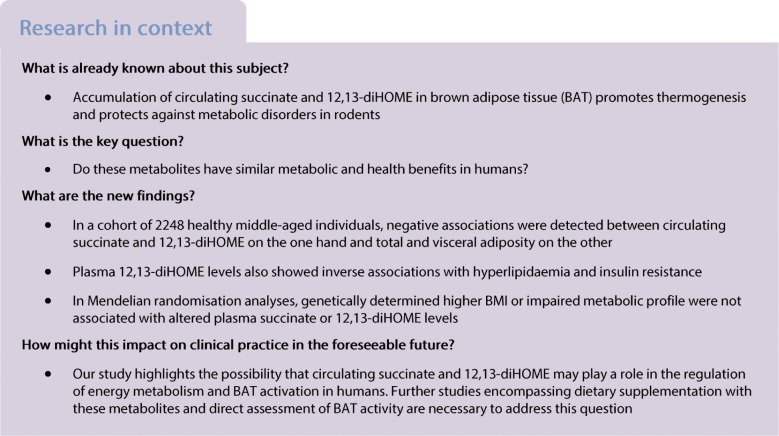



## Introduction

Obesity is associated with the development of type 2 diabetes and cardiovascular disease. The rising prevalence of obesity has focused attention on the discovery of safe and effective therapeutics promoting weight-loss and/or a healthy metabolic profile. Mammals possess two adipocyte types. White adipocytes function to store excess energy whereas brown adipocytes dissipate energy as heat and serve to maintain body temperature via non-shivering thermogenesis. In addition to thermogenesis, activation of brown adipose tissue (BAT) in rodents was shown to ameliorate hyperlipidaemia, promote glucose tolerance and insulin sensitivity, and protect against obesity and atherosclerosis [1–3]. In adult humans BAT activity was also shown to be inversely correlated with total and visceral adiposity and fasting plasma glucose [4, 5]. Chronic cold exposure and β3-adrenergic agonist treatment further led to BAT activation concomitant with an improvement in insulin sensitivity and/or a reduction in body fat mass [6–10]. These findings highlight that pharmacological induction of BAT thermogenesis in humans may ameliorate obesity and related metabolic disorders. However, pharmacologic targeting of β3 adrenoceptors has thus far demonstrated limited clinical efficacy and/or unacceptable side-effects. Hence, discovering novel BAT activation pathways is an important research focus.

Succinate is a tricarboxylic acid cycle (TCA) intermediate which can also exit mitochondria and function in the circulation. Succinate was recently identified as a systemic signal promoting BAT thermogenesis in rodents [11]. Specifically, circulating succinate was shown to increase following cold exposure in response to muscle shivering and selectively taken up by BAT leading to acute activation of thermogenesis. Pharmacological elevation of plasma succinate drove BAT thermogenesis with resultant protection against diet-induced obesity and glucose intolerance. Using a global lipidomic analysis, another landmark study identified 12,13-dihydroxy-9Z-octadecenoic acid (12,13-diHOME) as a lipid species increased in the circulation of humans and mice following cold exposure [12]. Injection of 12,13-diHOME in rodents acutely activated BAT NEFA uptake leading to enhanced cold tolerance. Chronic treatment of diet-induced obese mice with 12,13-diHOME led to a reduction in circulating triacylglycerol. In a follow-up study, plasma 12,13-diHOME levels were also shown to increase in response to an acute bout of exercise in humans and mice [13]. Acute treatment of mice with 12,13-diHOME led to increased skeletal muscle NEFA uptake and oxidation. Prompted by these findings, we set out to investigate the relevance of plasma succinate and 12,13-diHOME in the regulation of energy metabolism in a large cohort of healthy volunteers.

## Methods

### Study design, study population, biochemistry and dual-energy x-ray absorptiometry

This was a cross-sectional study. The Oxford BioBank (OBB) comprises a randomised, age-stratified sample obtained from Oxfordshire and the Thames Valley in the UK [14]. The Thames Valley Primary Care Agency has enabled random recruitment by providing lists of Oxfordshire residents registered with a local general practitioner and aged 30–50 years. An invitation letter along with the study information and response sheet were sent to all participants. Individuals who expressed willingness to enrol in the OBB were contacted by telephone or email, in order to convey a brief overview of the study aims and objectives, by trained research nurses. Individuals with a previous diagnosis of myocardial infarction or heart failure currently on treatment; untreated malignancy; other ongoing systemic diseases, and pregnant women were excluded from participation. The OBB recruitment began in 1999 and included 7640 individuals (4316 women and 3324 men) as of October 2016. Information regarding weekly physical activity was obtained using a validated questionnaire based on which participants were classified as sedentary, moderately or vigorously active [14]. Venous plasma samples were obtained after an overnight fast and analysed for glucose, cholesterol and triacylglycerol using Instrumentation Laboratory test kits (Werfen, Warrington, UK), HDL- and LDL-cholesterol using Randox kits (Randox Laboratories, Crumlin, Northern Ireland, UK) and insulin using Millipore Human Insulin specific radioimmunoassays (Millipore, Watford, UK). Whole-body dual-energy x-ray absorptiometry (DXA) was performed using a Lunar iDXA (GE Medical Systems, Madison, WI, USA) by trained research nurses. Acquired images were processed using enCORE v14.1 software. All studies were approved by the Oxfordshire Clinical Research Ethics Committee and all volunteers gave written, informed consent.

### Metabolomics

Plasma succinate and 12,13-diHOME levels were quantified by ultra-performance LC coupled with tandem MS (DiscoveryHD4 platform, Metabolon) as described [15]. Funding was available to undertake metabolomic screening in 2,248 participants. Individuals were selected based on their DXA-determined fat distribution with the aim of generating metabolomic data from a group of individuals with a wide range of regional adiposity representative of the general population. Raw area counts were normalised to the median value of the run day to correct for inter-day variation in the measurements. After log_10_ transformation, outlier values (>4 SDs from the mean) were removed.

### Statistical methods

Descriptive data are presented as mean and SD for normally distributed variables, median and interquartile range for skewed variables, and frequency (percentages) for categorical variables. Missing data for insulin (*n* = 1) and visceral fat mass (*n* = 6) were excluded from analysis. An age- and sex-adjusted linear regression model was used to examine the association between succinate and 12,13-diHOME and phenotypic traits. Outcome measurements were z-transformed (mean = 0, SD = 1) and effect sizes are presented as β-coefficients and 95% CI for standardised outcomes. Analyses were undertaken using Stata version 15.1 (College Station, TX, USA).

### Mendelian randomisation

We conducted a two-sample Mendelian randomisation (MR) study to investigate the relation between multiple traits for adiposity (BMI and WHR adjusted for BMI), fasting glucose homeostasis (fasting glucose, fasting insulin, homeostatic model assessment for insulin resistance), fasting lipids (triacylglycerols, LDL-cholesterol, HDL-cholesterol), and succinate and 12,13-diHOME plasma levels. For the anthropometric traits, we used 293 independent variants (pairwise *r*^2^ < 0.001) for BMI (based on *N* = 681,275) [16], and 282 independent variants (pairwise *r*^2^ < 0.001) for WHR adjusted for BMI (based on *N* = 694,649) [17]. For fasting glucose homeostasis traits, we used the findings from European-based analyses of the Meta-Analyses of Glucose and Insulin-related traits Consortium (MAGIC) (based on *N* of up to 76,558 individuals without diabetes mellitus) [18]. For the fasting lipid traits, we used the findings of the Global Lipids Genetics Consortium (GLGC; based on *N* of up to 94,595) [19]. As outcome data, we used summary statistics of genome wide association study (GWAS) analyses on OBB imputation level SNP data (*N* = 2117) using SNPTEST v2.5.2 software (https://mathgen.stats.ox.ac.uk/genetics_software/snptest/snptest.html) [20]. Succinate and 12,13-diHOME levels were adjusted for the first four genetic principal components and age and the residuals were rank-based inverse normally transformed for men and women separately. We used the well-established methods for two-sample MR analyses; namely inverse-variance weighted (IVW), MR-Egger and median-weighted analyses (MWA), as previously described [21]. These latter two methods for two-sample MR analyses were specifically used to test and/or take into account potential bias due to the presence of directional pleiotropy [22, 23] Analyses were conducted using the TwoSampleMR package implemented in R (v3.5.1) statistical software [24].

## Results

### Study population characteristics

The study participant characteristics are shown in Table 1. By design equal numbers of men and women were included in the study (*n* = 1,124 of each sex). The age (mean ± SD) of the study volunteers was 41.3 ± 5.9 years. Eighty per cent of participants were overweight (25 ≤ BMI <30 kg/m^2^); the mean ± SD BMI of the cohort was 26.1 ± 4.6 kg/m^2^. There were missing data for insulin in one individual and visceral fat mass measurements were uninterpretable and thus excluded in six people. Women had higher plasma 12,13-diHOME levels vs men (*p* = 0.003). No sex difference in plasma succinate levels was detected.

**Table 1:** Characteristics of the study population

	Total	Women	Men
	(*n* = 2248)	(*n* = 1124)	(*n* = 1124)
Female, *n* (%)	1124 (50)		
Age (years)	41.3 (5.9)	41.4 (5.9)	41.3 (5.9)
Height (cm)	172.4 (9.3)	165.6 (6.3)	179.2 (6.6)
Weight (cm)	77.6 (16.1)	69.8 (13.9)	85.6 (14.3)
BMI (kg/m^2^)	26.1 (4.6)	25.4 (5.0)	26.6 (4.0)
Systolic BP (mmHg)	122 (14)	117 (74)	127 (12)
Diastolic BP (mmHg)	76 (9)	74 (10)	79 (9)
Biochemistry			
Glucose (mmol/l)	5.3 (0.7)	5.1 (0.5)	5.4 (0.7)
Insulin (pmol/l)^a^	79.2 (59.1–107.6)	74.3 (56.3–101.4)	83.3 (63.2–113.9)
HOMA-IR^a^	2.6 (1.9, 3.7)	2.4 (1.8, 3.4)	2.9 (2.1, 4.0)
Triacylglycerol (mmol/l)^a^	1.0 (0.7, 1.4)	0.8 (0.6, 1.1)	1.2 (0.8, 1.7)
Total cholesterol (mmol/l)	5.1 (1.0)	5.0 (1.0)	5.3 (1.1)
HDL-cholesterol (mmol/l)	1.3 (0.4)	1.5 (0.4)	1.2 (0.3)
NEFA (μmol/l)^a^	431 (299–593)	477 (337–651)	392 (271–521)
Glycerol (μmol/l)^a^	45 (31–65)	56 (39–76)	36 (26–52)
β-OH butyrate (μmol/l)^a^	56 (34–104)	62 (36–119)	50 (33–86)
Lactate (mmol/l)^a^	0.7 (0.6–1.0)	0.7 (0.5–0.9)	0.8 (0.6–1.1)
12,13-diHOME^a^	0.9 (0.5–1.2)	0.9 (0.6–1.3)	0.9 (0.5–1.2)
Succinate^a^	1.0 (0.8–1.2)	1.0 (0.9–1.2)	1.0 (0.9–1.3)
DXA body composition			
Total fat mass (kg)^a^	22.5 (17.2–28.6)	22.9 (17.8–29.5)	22.0 (16.9–27.8)
Total fat mass (%)	31.9 (8.4)	27.7 (6.9)	36.2 (7.8)
Visceral fat (kg)^a^	0.5 (0.2–1.2)	0.3 (0.1–0.6)	0.9 (0.5–1.6)
Total lean mass (kg)^a^	48.6 (41.2–57.8)	41.4 (38.1–44.8)	57.6 (53.2–62.6)

Data are mean (SD) unless otherwise indicated

^a^Median (interquartile range)

### Associations between circulating succinate and 12,13-diHOME levels and body composition and systemic metabolism

In a sex- and age-adjusted linear regression model, plasma succinate was negatively associated with DXA-determined total and visceral adiposity. A one-SD increase in plasma succinate concentration was associated with a 0.15 SD (95% CI 0.28 to 0.03) lower total fat mass and a 0.11 SD (95% CI 0.22 to 0.004) lower visceral fat mass; equivalent to 1.5 kg and 89 g respectively. In contrast, positive relationships were observed between systemic succinate and plasma HDL-cholesterol, NEFA, glycerol and lactate levels (Table 2). The associations between circulating succinate concentration and visceral fat mass and HDL-cholesterol became non-significant after additional adjustment for total fat mass percentage, physical activity or both (electronic supplementary material [ESM] Tables 1–3). Adjustment for physical activity also abolished the association between plasma succinate and total fat mass.

**Table 2:** Age- and sex-adjusted associations between succinate levels and metabolic and body composition markers

	12,13-diHOME		Succinate	
	β (95% CI)	*p* value	β (95% CI)	*p* value
z-Weight	−0.05 (−0.11, 0.01)	0.09	−0.10 (−0.21, 0.01)	0.07
z-BMI	−0.00 (−0.15, 0.02)	0.009	−0.12 (−0.24, 0.004)	0.06
z-Systolic BP	−0.04 (−0.10, 0.03)	0.27	0.08 (−0.03, 0.20)	0.15
z-Diastolic BP	−0.08 (−0.14, −0.01)	0.018	0.05 (−0.07, 0.17)	0.41
Biochemistry				
z-Glucose	−0.12 (−0.19, −0.06)	0.0002	−0.10 (−0.22, 0.02)	0.09
z-Insulin	−0.09 (−0.16, −0.02)	0.008	−0.07 (−0.19, 0.06)	0.28
z-HOMA-IR	−0.10 (−0.17, −0.03)	0.003	−0.08 (−0.20, 0.04)	0.19
z-Triacylglycerol	−0.10 (−0.17, −0.04)	0.002	−0.06 (−0.18, 0.06)	0.30
z-Total cholesterol	−0.06 (−0.12, 0.01)	0.07	0.04 (−0.08, 0.16)	0.50
z-HDL-cholesterol	0.14 (0.08, 0.20)	1.4 × 10^−5^	0.13 (0.02, 0.25)	0.023
z-NEFA	0.15 (0.08, 0.21)	1.1 × 10^−5^	0.19 (0.07, 0.31)	0.002
z-Glycerol	0.15 (0.09, 0.21)	1.1 × 10^−6^	0.29 (0.17, 0.41)	1.2 × 10^−6^
z-β-OH butyrate	0.12 (0.06, 0.19)	1.5 × 10^−4^	0.12 (−0.01, 0.24)	0.07
z-Lactate	−0.05 (−0.12, 0.01)	0.10	0.21 (0.08, 0.33)	0.001
DXA body composition				
z-Total fat mass	−0.08 (−0.15, −0.01)	0.021	−0.15 (−0.27, −0.03)	0.015
z-Total fat percentage	−0.11 (−0.16, −0.04)	0.001	−0.17 (−0.27, −0.06)	0.002
z-Visceral fat	−0.09 (−0.15, −0.03)	0.004	−0.11 (−0.22, −0.004)	0.041
z-Total lean mass	0.001 (−0.04, 0.04)	0.95	−0.002 (−0.08, 0.08)	0.97

Linear regression model adjusted for age and sex, with z-transformed values

β-values represent an SD change in 12,13-diHOME/succinate with corresponding SD change in metabolic and body composition traits

As shown in Table 2, plasma 12,13-diHOME exhibited an inverse relationship with total adiposity, visceral adiposity and an adverse metabolic profile. These associations were stronger in women (data not shown). A one-SD increase in plasma 12,13-diHOME concentration was associated with a 0.08 SD (95% CI 0.15 to 0.01) decrease in total fat mass, a 0.09 SD (95% CI 0.15 to 0.03) reduction in visceral fat mass, a 0.10 SD (95% CI 0.16 to 0.02) reduction in fasting plasma insulin and 0.10 SD (95% CI 0.17 to 0.04) decrease in plasma triacylglycerols; equivalent to 0.75 kg, 68 g, 5.1 pmol/l and 0.082 mmol/l respectively. Plasma 12,13-diHOME also displayed positive associations with HDL-cholesterol, NEFA, glycerol and β-hydroxybutyrate (β-OH butyrate). The associations between circulating 12,13-diHOME concentration and visceral adiposity, as well as plasma biochemistry, were attenuated, but with the exception of fasting insulin, remained significant after accounting for total fat mass percentage as a covariate in the linear regression model (ESM Table 1). Adjustment for physical activity or both fat mass percentage and physical activity led to similar results (ESM Tables 2, 3).

### MR of BMI and circulating succinate and 12,13-diHOME levels

Based on IVW, we did not find evidence that genetically determined obesity, elevated fasting glucose, raised fasting insulin and HOMA-IR, or hyperlipidaemia were associated with altered plasma succinate or 12,13-diHOME levels (Table 3). However, a genetically increased WHR adjusted for BMI was associated with both higher plasma succinate (0.34 SD; *p* value = 0.003) and higher plasma 12,13-diHOME concentrations (0.33 SD; *p* value = 0.007). Sensitivity analyses using MR-Egger and weighted median methods showed similar effect estimates (results not shown) and the intercept of the MR-Egger regression test did not deviate from zero in any of the analyses (Table 3), suggesting that these results were unlikely to be due to bias caused by the presence of directional pleiotropy.

**Table 3:** MR analyses on blood succinate and 12,13-diHOME levels in the Oxford BioBank

		Succinate			12,13-diHOME		
	*n* instruments	Beta (SE)	*p* value	MR-Egger test	Beta (SE)	*p* value	MR-Egger test
Adiposity							
BMI	293	0.15 (0.10)	0.11	0.36	0.08 (0.10)	0.41	0.79
WHRadjBMI	282	0.34 (0.12)	0.003	0.96	0.33 (0.13)	0.009	0.22
Glucose homeostasis							
Glucose	11	0.54 (0.31)	0.08	0.90	0.00 (0.47)	1.00	0.07
Insulin^a^	2	1.14 (1.21)	0.35	NA	1.53 (0.95)	0.11	NA
HOMA-IR^a^	2	0.98 (0.14)	0.39	NA	1.39 (0.86)	0.11	NA
Lipid levels							
Triacylglycerols	35	−0.12 (0.11)	0.26	0.58	−0.11 (0.13)	0.39	0.75
LDL-cholesterol	43	0.07 (0.09)	0.45	0.53	−0.07 (0.08)	0.42	0.80
HDL-cholesterol	60	−0.05 (0.11)	0.65	0.81	−0.14 (0.11)	0.22	0.86

Results are presented as beta estimates with SE

Results were retrieved using the IVW method with the assumption of no bias by directional pleiotropy

The MR-Egger test was used to detect the presence of potential directional pleiotropy

^a^It was not possible to conduct the MR-Egger test in the presence of only two genetic instruments

WHRadjBMI, WHR adjusted for BMI

## Discussion

Our study provides novel insights, as well as confirming previous cross-sectional data from small cohorts, about the association of plasma succinate and 12,13-diHOME with adiposity and metabolic traits in humans. Our data highlight a potential role for both metabolites in the regulation of energy balance, systemic metabolism and BAT activation in humans, thereby extending the results of recent landmark studies demonstrating that circulating succinate and 12,13-diHOME promote brown adipocyte thermogenesis and protect against metabolic disorders in rodents [11, 12]. Nonetheless, despite—to our knowledge—this being the largest human series to date to directly examine the associations of these metabolites with body composition and plasma biochemistry, our study has limitations. Namely, we did not investigate the effects of pharmacological supplementation with succinate or 12,13-diHOME on adiposity, metabolic profile or BAT activation; nor did we measure BAT activity or systemic markers thereof (e.g. FGF21) in our study participants. As such our results should be interpreted with caution. Additionally, physical activity was determined using a questionnaire rather than accelerometer devices which are the gold standard. Finally, we did not have information as to whether study participants had been physically active in the few hours prior to blood extraction. This may be another limitation of our study given that an acute spurt of exercise has been shown to increase both plasma succinate and 12,13-diHOME levels in humans [13, 23]. However, all blood sampling was undertaken in the fasted state in the early morning hence it is unlikely that this was the case. Furthermore, any noise introduced in our dataset by physical activity prior to blood sampling is likely to have diluted rather than strengthened the observed associations due to ‘measurement error’.

In addition to acting as a TCA intermediate, succinate is used as a food additive and dietary supplement. Succinate might also be released into the circulation by gut commensal bacteria which produce large amounts of this metabolite through fermentation of dietary fibre [25]. The association between circulating succinate levels and cardiometabolic disease was previously investigated only in small series. In a cohort of 91 participants, plasma succinate was higher in obese than in lean individuals and those with type 2 diabetes (*n* = 20) vs BMI-matched normoglycaemic participants [26]. Similarly, van Diepen et al found elevated plasma succinate levels in 58 participants with type 2 diabetes compared with 76 healthy control participants [27]. In contrast, another study found no difference in fasting and postprandial serum succinate between 23 participants with type 2 diabetes and 15 control participants [28]. Similarly, no differences in serum succinate were detected between individuals with (*n* = 172) and without (*n* = 61) coronary artery stenosis [29]. Herein, we extend these findings by demonstrating an inverse association between plasma succinate and DXA-determined total and visceral adiposity, as well as a positive association with HDL-cholesterol, NEFA, glycerol and lactate levels. The positive association between plasma succinate and NEFA, as well as glycerol concentrations, was unexpected given that succinate acting via succinate receptor 1 (SUCNR1) was previously shown to inhibit adipocyte lipolysis [30]. This finding is likely to reflect increased succinate production from TCA metabolism by oxidation of carbons derived from NEFA. A similar mechanism is also likely to explain the positive relationship between systemic succinate and lactate levels as glucose was shown to feed the TCA cycle via circulating lactate [31]. We were unable to examine the association between plasma succinate levels and type 2 diabetes status as our study population was composed solely of healthy participants. Of note, with the exception of total fat mass percentage, the associations between circulating succinate and reduced adiposity, as well as increased HDL-cholesterol, became non-significant after adjustment for physical activity. These results suggest that, in addition to potentially having direct effects on systemic metabolism, plasma succinate may also serve as a surrogate marker of cardiorespiratory fitness; indeed exercise is known to lead to elevated plasma succinate through increased muscle contraction [32].

12,13-diHOME is produced by oxidation of linoleic acid, an essential fatty acid which is abundant in many nuts and fatty seeds, followed by hydrolysis which is catalysed by soluble epoxide hydrolases. The latter are encoded by four genes, *EPHX1-4*. Like succinate, gut commensal bacteria might also contribute to plasma 12,13-diHOME levels [33]. A potential link between 12,13-diHOME and metabolic traits was first reported by Schuchardt et al [34] who showed that the serum concentration of this lipokine was lower in 20 normolipidaemic vs 20 hyperlipidaemic middle-aged men. Subsequently, plasma 12,13-diHOME levels were shown to be strongly and negatively correlated with BMI, HOMA-IR and fasting plasma triacylglycerols in 55 predominantly female participants, including ten with type 2 diabetes [12]. Associations between 12,13-diHOME and BMI and triacylglycerols were confirmed in a follow-up study comprising 39 healthy individuals which also demonstrated a correlation between 12,13-diHOME and cardiovascular fitness [13]. Our study broadly confirms these findings. However, in our cohort the associations between systemic 12,13-diHOME levels and anthropometric and metabolic traits were weaker than those previously reported. Also, contrary to these earlier studies, which found no significant associations between 12,13-diHOME and HDL-cholesterol and/or fasting glucose, these two metabolites exhibited statistically robust relationships with 12,13-diHOME in the OBB. These differences are probably due to the large size and composition of our study population which comprised healthy individuals with a more narrow age and BMI range than those reported in earlier studies. Due to the relatively large size of our study cohort we were additionally able to show that circulating 12,13-diHOME levels were higher in women than men. The sexual dimorphism in systemic 12,13-diHOME concentration had not been observed in earlier studies [12, 13]. We also detected strong positive associations between plasma 12,13-diHOME and NEFA, as well as glycerol levels. We interpret these data as being consistent with the release of 12,13-diHOME from adipocytes into the systemic circulation consequent to fasting-induced lipolysis. In this respect, Lynes et al detected a high concentration of 12,13-diHOME in mouse white adipose tissue and, whilst the concentration of this lipokine has not been determined in human fat, based on RNAseq data from GTEx (https://gtexportal.org) *EPHX1* and *2* are both highly expressed in this tissue. Plasma 12,13-diHOME also correlated positively with circulating β-OH butyrate levels, a result in keeping with elevated fasting-induced lipolysis providing increased NEFA substrates for hepatic ketogenesis. An acute bout of moderate exercise has been shown to cause a pronounced increase in circulating 12,13-diHOME levels in humans. Active male participants also had significantly higher baseline 12,13-diHOME levels compared with their sedentary counterparts [13]. In this regard, it is noteworthy that the associations of circulating 12,13-diHOME levels with body composition and metabolic profile, whilst attenuated, remained significant after adjustment for physical activity. These data suggest that this lipokine has direct effects on systemic metabolism, possibly by promoting BAT activity.

MR analyses did not support a causal influence of increased obesity in reducing succinate or 12,13-diHOME plasma levels. Whilst these results may be due to insufficient statistical power (the OBB dataset with available metabolomic data was small [*n* = 2248]), any undetected effects are likely to be very small. Together with the observational data presented herein, these findings suggest that higher circulating levels of succinate and 12,13-diHOME may be causally linked to, rather than being a consequence of, reduced fat mass accumulation. Unfortunately, we were unable to directly test this hypothesis using MR owing to the lack of available genetic instruments associated with plasma succinate or 12,13-diHOME levels. Similarly, we discovered no evidence that a genetically determined abnormal metabolic profile modulates systemic levels of succinate or 12,13-diHOME although, in the case of raised fasting glucose, insulin and HOMA-IR, this may have been due to the lack of sufficient genetic instruments and low statistical power. On the other hand we did find that a genetic predisposition to upper-body fat distribution was associated with higher circulating levels of both metabolites. We speculate that this is a consequence of the increased spontaneous lipolysis associated with central obesity [35].

In conclusion, we provide evidence that circulating succinate and 12,13-diHOME may play a role in the regulation of energy balance and systemic metabolism in humans. Further studies involving direct assessment of physical and BAT activity, dietary supplementation and bidirectional MR are necessary to examine the potential health benefits of these proposed thermogenic metabolites.

## Electronic supplementary material

ESM Tables (PDF 171 kb)

## Data Availability

Data relating to the current study are available on request from the corresponding authors CC and FK.

## Abbreviations

BAT: Brown adipose tissue
β-OH butyrate: β-Hydroxybutyrate
12,13-diHOME: 12,13-Dihydroxy-9Z-octadecenoic acid
DXA: Dual-energy x-ray absorptiometry
IVW: Inverse-variance weighted
MR: Mendelian randomisation
OBB: Oxford BioBank
TCA: Tricarboxylic acid cycle
