# Supplementary material for: The proposed systemic thermogenic metabolites succinate and 12,13-diHOME are inversely associated with adiposity and related metabolic traits: evidence from a large human cross-sectional study
Source: Diabetologia. 2019 Jul 15;62(11):2079–87. doi: 10.1007/s00125-019-4947-5 (PMC6805813; doi:10.1007/s00125-019-4947-5)
Supplement: Supplementary file 1 — (PDF 171 kb) [file 125_2019_4947_MOESM1_ESM.pdf]

ESM Table 1. Age-, sex-, and total fat mass-adjusted associations between succinate levels and metabolic and body composition markers.

	12,13 diHOME		Succinate	
	β(95%CI)	P value	β(95%CI)	P value
z-Systolic BP	-0.02 (-0.08, (0.04)	0.54	0.11 (0.004, 0.22)	0.04
z-Diastolic BP	-0.05 (-0.11, 0.01)	0.09	0.10 (-0.01, 0.21)	0.09
Biochemistry				
z-Glucose	-0.10 (-0.17, -0.04)	0.001	-0.07 (-0.18, 0.05)	0.27
z-Insulin	-0.05 (-0.11, 0.01)	0.08	0.01 (-0.10, 0.11)	0.92
z-HOMA IR	-0.06 (-0.12, -0.004)	0.035	-0.01 (-0.12, 0.10)	0.85
z-Triglyceride	-0.08 (-0.14, -0.02)	0.012	-0.02 (-0.13, 0.10)	0.77
z-Total Cholesterol	-0.05 (-0.11, 0.02)	0.13	0.06 (-0.06, 0.18)	0.31
z-HDL-cholesterol	0.11 (0.05, (0.17)	1.8×10^{-04}	0.08 (-0.03, 0.18)	0.15
z-NEFA	0.16 (0.09, 0.22)	3.9×10^{-06}	0.20 (0.08, 0.32)	0.001
z-Glycerol	0.17 (0.11, 0.23)	0.006	0.32 (0.21, 0.44)	2.4×10^{-8}
z-β-hydroxy butyrate	0.12 (0.05, 0.18)	3.1×10^{-05}	0.10 (-0.02, 0.23)	0.11
z-Lactate	-0.04 (-0.09, 0.03)	0.25	0.24 (0.12, 0.36)	8.4×10^{-6}
DXA-body composition				
z-Visceral fat	-0.03 (-0.06, (0.01)	0.12	-0.003 (-0.07, 0.06)	0.94
z-Total lean mass	0.02 (-0.01, (0.06)	0.23	0.04 (-0.03, 0.11)	0.25

Linear regression model adjusted for age, sex and total fat mass. β-values represent a S.D. change in 12,13-diHOME/Succinate with corresponding S.D. change in metabolic and body composition traits.

ESM Table 2. Age- and sex and physical activity-adjusted associations between succinate levels and metabolic and body composition markers

	12,13 diHOME		Succinate	
	β (95%CI)	P value	β (95%CI)	P value
z-Weight	-0.03 (-0.09, 0.02)	0.23	-0.07 (-0.18, 0.03)	0.17
z-BMI	-0.06 (-0.12, -0.002)	0.043	-0.09 (-0.21, 0.03)	0.14
z-Systolic blood pressure	0.04 (-0.10, -0.01)	0.13	0.09 (-0.02, 0.20)	0.11
z-Diastolic blood pressure	-0.08 (-0.14, -0.02)	0.01	0.07 (-0.05, 0.18)	0.24
Biochemistry				
z-Glucose	-0.10 (-0.16, -0.04)	0.001	-0.09 (-0.20, 0.03)	0.16
z-Insulin	-0.07 (-0.13, -0.01)	0.027	-0.03 (-0.15, 0.09)	0.58
z-HOMA IR	-0.08 (-0.14, -0.02)	0.013	-0.05 (-0.17, 0.07)	0.43
z-Triglyceride	-0.08 (-0.14, -0.02)	0.007	-0.04 (-0.15, 0.08)	0.54
z-Total Cholesterol	-0.06 (-0.11, 0.01)	0.07	0.05 (-0.06, 0.17)	0.39
z-HDL-cholesterol	0.11 (0.05, 0.16)	2.9×10^{-03}	0.10 (-0.01, 0.21)	0.08
z-NEFA	0.11 (0.05, 0.18)	4.2×10^{-03}	0.19 (0.07, 0.31)	0.002
z-Glycerol	0.15 (0.09, 0.21)	5.8×10^{-07}	0.30 (0.18, 0.42)	4.73×10^{-7}
z-β-hydroxy butyrate	0.12 (0.06, 0.18)	2.6×10^{-03}	0.10 (-0.02, 0.22)	0.11
z-Lactate	-0.05 (-0.22, 0.01)	0.12	0.21 (0.09, 0.33)	0.001
DXA-body composition				
z-Total fat mass	-0.05 (-0.11, 0.01)	0.13	-0.11 (-0.23, 0.01)	0.06
z-Total fat percentage	-0.06 (-0.11, -0.009)	0.022	-0.13 (-0.23, -0.03)	0.014
z-Visceral fat	-0.07 (-0.12, -0.01)	0.017	-0.08 (-0.18, 0.03)	0.16
z-Total lean mass	-0.002 (-0.04, 0.03)	0.92	-0.002 (-0.08, 0.07)	0.96

Linear regression model adjusted for age and sex. β-values represent a SD change in 12,13-diHOME/Succinate with corresponding SD change in metabolic and body composition traits.

ESM Table 3. Age-, sex-, physical activity and total fat mass-adjusted associations between succinate levels and metabolic and body composition markers.

	12,13 diHOME		Succinate	
	β (95%CI)	P value	β (95%CI)	P value
z-Systolic BP	-0.03 (-0.09, 0.02)	0.28	0.12 (0.01, 0.23)	0.04
z-Diastolic BP	-0.06 (-0.12, -0.00)	0.048	0.11 (-0.003, 0.22)	0.06
Biochemistry				
z-Glucose	-0.09 (-0.15, -0.03)	0.004	-0.06 (-0.17, 0.06)	0.35
z-Insulin	-0.05 (-0.10, 0.01)	0.13	0.02 (-0.09, 0.13)	0.74
z-HOMA IR	-0.05 (-0.11, 0.004)	0.07	0.002 (-0.11, 0.12)	0.96
z-Triglyceride	-0.06 (-0.12, -0.006)	0.03	0.002 (-0.11, 0.11)	0.97
z-Total Cholesterol	-0.04 (-0.10, 0.02)	0.15	0.08 (-0.04, 0.19)	0.20
z-HDL-cholesterol	0.09 (0.03, 0.14)	0.002	0.06 (-0.05, 0.17)	0.27
z-NEFA	0.12 (0.06, 0.18)	1.2×10^{-05}	0.21 (0.08, 0.33)	0.001
z-Glycerol	0.17 (0.11, 0.23)	3.6×10^{-08}	0.33 (0.21, 0.44)	2.4×10^{-8}
z-β-hydroxy butyrate	0.11 (0.05, 0.18)	3.9×10^{-05}	0.09 (-0.02, 0.21)	0.13
z-Lactate	-0.04 (-0.09, 0.02)	0.24	0.24 (0.12, 0.36)	8.4×10^{-6}
DXA-body composition				
z-Visceral fat	-0.02 (-0.06, 0.02)	0.26	0.01 (-0.06, 0.09)	0.73
z-Total lean mass	0.004 (-0.03, 0.04)	0.82	0.01 (-0.06, 0.08)	0.76

Linear regression model adjusted for age, sex and total fat mass. β-values represent a S.D. change in 12,13-diHOME/Succinate with corresponding S.D. change in metabolic and body composition traits.
